# Oligometastatic Adenocarcinoma of the Lung: A Therapeutic Opportunity for Long-Term Survival

**DOI:** 10.7759/cureus.409

**Published:** 2015-12-16

**Authors:** Mark Yarchoan, Michael Lim, Julie R Brahmer, David Ettinger

**Affiliations:** 1 The Sidney Kimmel Comprehensive Cancer Center, Johns Hopkins; 2 Neurosurgical Oncology, Johns Hopkins

**Keywords:** oligometastatic, non-small cell lung cancers (nsclc), abscopal effect, brain metastasis, lung cancer

## Abstract

We report a case of oligometastatic non-small-cell lung cancer (NSCLC) in a 60-year-old male that was treated with both local and systemic therapies with an exceptional response to therapy. This case provides evidence that oligometastatic lung cancer, when treated with curative intent, may be an opportunity for long-term survival in select patients.

## Introduction

Stage IV metastatic lung cancers are generally thought to be incurable, and the mainstay of treatment is palliative systemic therapy. A distinct subset of metastatic lung cancer is the “oligometastatic state”, which is characterized by the presence of a limited number of metastasis in a limited number of regional sites. Whether local therapy improves progression-free survival and overall survival in such cases remains a matter of controversy.

Here, we present the case of a 60-year-old gentleman who presented with oligometastatic non-small-cell lung cancer (NSCLC) who was treated with both local and systemic therapies and experienced an exceptional response to therapy. Informed consent for the publication of this case presentation was obtained from the patient.

## Case presentation

**Figure 1:**
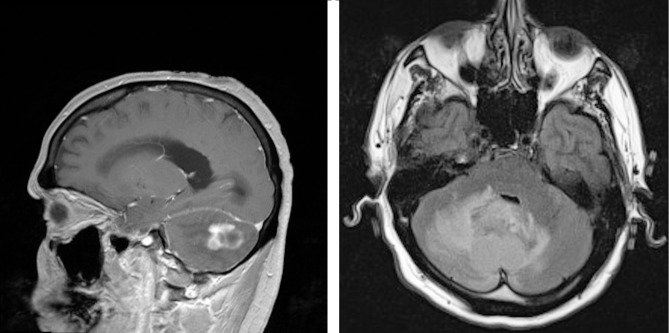
MRI of the brain revealed a 2.6 cm cerebellar lesion.

In July of 2009, patient JR, an active smoker with an 80 pack-year smoking history, presented to the emergency room of our hospital with several weeks of dizziness, headaches, nausea, and vomiting. Magnetic resonance imaging (MRI) performed upon admission revealed a 2.6 x 1.6 x 2.3 cm multilobulated cerebellar lesion (Figure 1). Informed patient consent was obtained. Emergent resection confirmed a pathologic diagnosis of adenocarcinoma, with immunostaining suggestive of a lung primary (positive for CK7, TTF-1, and Napsin-A, and negative for CK20). The patient was subsequently treated with whole brain radiation and localized stereotactic radiosurgery (SRS).

**Figure 2:**
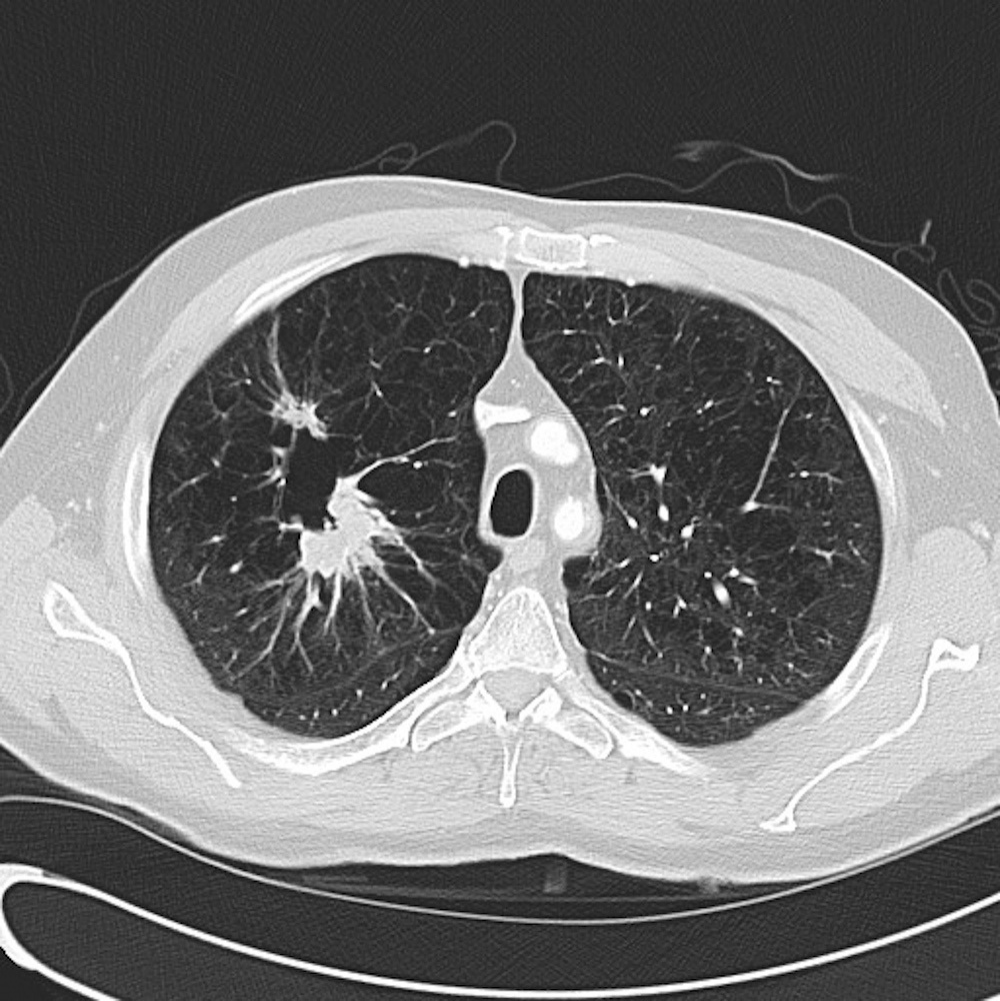
CT scan of the chest demonstrated two spiculated masses in the right upper lobe of the lung.

A postoperative computerized tomography (CT) scan of his chest showed two spiculated masses in the right upper lobe with additional small nodules in the right upper lobe and along the right minor fissures (Figure 2). These lesions showed intense FDG uptake on positron emitted tomography (PET)/CT, but there was no evidence of distant metastasis, except for the cerebellar mass that had already been resected. Several months later, the patient underwent a flexible bronchoscopy, mediastinoscopy, right thoracotomy, and an uncomplicated right upper lobe lobectomy, which confirmed that the patient had primary adenocarcinoma of the lung with distant metastasis to the brain. Pathology from his lobectomy revealed a 3.1 cm tumor with a separate satellite lesion in the right upper lobe and one positive hilar node. The tumor was KRAS and epidermal growth factor receptor (EGFR) wild-type. From December 2009 through February 2010, he received a total of four cycles of cisplatin and pemetrexed chemotherapy.

After the patient’s fourth cycle of chemotherapy, his basic chemistry labs demonstrated persistent hypercalcemia, concerning for persistent disease, so a repeat PET/CT was obtained. This restaging scan demonstrated a new small nodule with FDG uptake in the left adrenal gland, a pulmonary nodule in the left lower lobe, and another in the liver, all of which were concerning for metastatic disease. Additionally, a new 1.1 cm right frontal asymptomatic lesion was discovered on brain MRI, which the patient opted to treat with SRS in June 2010. Shortly after completing this treatment, a second new right parietal cortex lesion measuring 0.5 cm was observed on subsequent MRI, which was also treated with SRS.

After completion of stereotactic radiosurgery to these two separate brain lesions in July 2010, the patient re-presented to medical oncology for consideration of additional systemic therapy. However, a restaging CT scan at this point showed no evidence of recurrent disease. The previously observed adrenal, lung, and liver lesions were no longer seen. Because he had no measurable disease and felt well, he was observed off therapy. Since November of 2010, he has received no additional therapy, and multiple restaging scans have not shown any evidence of recurrent disease. At the time of this writing, he has had no evidence of recurrent disease for over five years.

## Discussion

The role of localized forms of treatment for patients with metastatic NSCLC remains a subject of considerable debate, and prospective data are lacking about whether localized therapies can affect overall survival in the metastatic setting. In order for cancer to metastasize, it has been proposed that cancer must be microscopically present in the systemic circulation in a leukemic-like state, suggesting that it can only benefit from systemic therapy. However, in 1995, Samuel Hellman and Ralph Weichselbaum proposed that there exists an intermediate state of metastatic disease, which he termed the “oligometastatic state” [1]. They proposed that in this intermediate stage, the number of metastatic tumors and the number of organs affected are limited and might, therefore, be amenable to localized forms of treatment. Since that time, localized therapies have been shown to have a role in several metastatic cancers, such as surgical resection of hepatic metastasis in colon cancer and radical nephrectomy in metastatic renal cell carcinoma [2].

In NSCLC, data from available, mostly small retrospective studies have demonstrated favorable disease-free intervals and improved overall survival in some patients receiving localized therapy to sites of oligometastatic disease [3-4]. However, prospective studies are lacking and it is it is unclear if the favorable survival times are a result of the localized treatment or to other factors, such as patient selection bias and the inherently indolent biology of oligometastatic disease. In retrospective studies, the survival of patients with oligometastatic NSCLC is more favorable than in those with extensive metastasis, even for patients who do not receive localized forms of treatment [5]. The subject of this case report presented with oligometastatic lung cancer and was treated aggressively with both systemic and localized therapy. In the absence of adequate randomized clinical trials to address the management of oligometastatic NSCLC, his exceptional response provides evidence that in at least a subset of patients with oligometastatic NSCLC, local therapy with curative intent rather than palliative treatment may be appropriate.

One of the most intriguing aspects of this case is the spontaneous regression of this patient’s nodules in the left adrenal gland, a pulmonary nodule in the left lower lobe, and the liver, following SRS for brain metastases. These small nodules were never biopsied and so it is not possible to conclude definitively that they were metastases of his NSCLC; however, the clinical context and radiographic appearance of the nodules is convincing. Their disappearance following radiosurgery may be an example of the abscopal effect, which describes distant effects of localized irradiation. Many demonstrations of the abscopal effect have been published, and although the precise mechanism(s) underlying the abscopal effect are not known, it has been proposed that radiosurgery can have distant anti-tumor effects through immune-mediated mechanisms [6].

## Conclusions

In conclusion, we present a case of oligometastatic NSCLC that was treated with both systemic and localized therapies. This patient’s exceptional response provides evidence that oligometastic lung cancer, when treated with curative intent, may be an opportunity for long-term survival in select patients. However, prospective randomized trials examining the outcomes of localized therapy to sites of oligometastatic disease in NSCLC are needed.
